# Prevalence and Associated Risk Factors of Tooth Wear

**DOI:** 10.31729/jnma.3644

**Published:** 2018-08-31

**Authors:** Deepti Shrestha, Prerisha Rajbhandari

**Affiliations:** 1 Department of Conservative Dentistry and Endodontics, Kathmandu Medical College and Teaching Hospital, Duwakot, Kathmandu, Nepal

**Keywords:** *basic erosive wear examination*, *risk factor*, *tooth wear*

## Abstract

**Introduction:**

Tooth wear is described as loss of hard tooth tissue with no occurrence of dental caries or trauma. Basic Erosive Wear Examination, a new scoring system, is a partial scoring system recording the most severely affected surface in a sextant and the cumulative score guides the management of the condition for the practitioner. The objectives of this study were to determine the prevalence of tooth wear and its association with its risk factors like gender, oral hygiene, diet, general health and life style.

**Methods:**

A cross-sectional study was done in 364 dental patients of Kathmandu Medical College. A questionnaire was filled by interview and Basic Erosive Wear Examination was done. The data so collected were entered in Statistical Package for Social Sciences. Descriptive statistical analysis and Chi-square tests were done at confidence interval of 95% and statistical significance was set at P=0.05.

**Results:**

The prevalence of tooth wear was 218 (60.1%) with no significant gender difference. A significant association was observed between tooth wear and age group (P<0.01), duration of brushing (P=0.05), method of brushing (P<0.01), swishing of soft drinks before swallowing (P<0.01) and tobacco chewing (P=0.02).

**Conclusions:**

The prevalence of tooth wear in the study population was high.

## INTRODUCTION

Tooth wear is described as loss of hard tooth tissue with no occurrence of dental caries or trauma.^1^ Tooth wear can be studied using various in vivo and in vitro studies. In vivo studies include clinical examination, photographs, indices, etc.^2^ Basic Erosive Wear Examination (BEWE), a new scoring system, is a partial scoring system recording the most severely affected surface in a sextant and the cumulative score guides the management of the condition for the practitioner.^3^

For proper management of tooth wear, it's diagnosis, etiology and risk factors must be evaluated.^1^ Studies to determine the prevalence tooth wear and associated risk factors can facilitate better assessment, planning of preventive measures, and carry out treatment more effectively.

The objectives of this study were to determine the prevalence of tooth wear and its association with its risk factors like gender, oral hygiene, diet, general health and life style.

## METHODS

This cross-sectional study was conducted at dental out patient department of Kathmandu Medical College and Teaching Hospital (KMCTH), Duwakot from August, 2017 to March 2018. Ethical approval was received from Institutional Review Committee, KMCTH. Patients aged 15–75 years, who gave written consent were included in the study and patients undergoing orthodontic treatment, extensive restoration, full coverage crown and multiple missing teeth were excluded.

With the estimated prevalence of 30%, with 95% confidence interval (CI), the following formula was used.


$$\text{n = }{\text{4pq/e}}^{\text{2}}$$

where p=prevalence of tooth wear that is 30%

q= 1-p

e= margin of error 5% (absolute error = 5%, a = 0.05).

Adding 5%, n=353

A pretested valid (a = 0.8) questionnaire with 15 closed ended multiple choice questions on demographics, oral hygiene, dietary factors, general health and life style was used. After obtaining the filled questionnaire through interview, a clinical examination was done using mouth mirror, periodontal probe and operating light and BEWE index given by Bartlett et al.^3^ was filled. Buccal/facial, occlusal/incisal and lingual/palatal surfaces of teeth in each sexant (excluding third molars) were examined and graded (Table 1). Only the surface with the highest score was recorded for each sextant and total score was calculated and risk level was classified (Table 2). The data so collected were entered in Statistical Package for Social Sciences (SPSS, released 2011, version 20.0. Armonk, NY:IBM corp.). Descriptive statistical analysis and Chi-square tests were done at confidence interval of 95% and statistical significance was set at P = 0.05.

**Table 1:** Criteria for grading erosive wear.

Score	Criteria for area
0	No erosive tooth wear
1	Initial loss of surface area
2	Distinct defect: Hard tissue loss <50 % of the surface area
3	Hard tissue loss ≥50%

**Table 2:** Risk levels as a guide to clinical management.

Risk level	Cumulative score	Management
None	≤ 2	Routine maintenance and observation Repeat at 3-year intervals
Low	3–8	Oral hygiene and dietary assessment, and advice, routine maintenance and observation Repeat at 2-year intervals
Medium	9–13	Oral hygiene and dietary assessment, and advice, identify the main etiological factor (s) for tissue loss and develop strategies to eliminate respective impacts Consider fluoridation measures or other strategies to increase the resistance of tooth surfaces Ideally, avoid the placement of restorations and monitor erosive wear with study casts, photographs, or silicone impressions Repeat at 6–12 month intervals
High	>14	Oral hygiene and dietary assessment, and advice, identify the main etiological factor (s) for tissue loss and develop strategies to eliminate respective impacts Consider fluoridation measures or other strategies to increase the resistance of tooth surfaces Ideally, avoid restorations and monitor tooth wear with study casts, photographs, or silicone impressions Especially in cases of severe progression consider special care that may involve restorations Repeat at 6–12 month intervals

## RESULTS

Total 364 patients (131 male and 233 female) participated in this study. The prevalence of tooth wear was 218 (60.1%) with no significant gender difference. The tooth wear increased with increasing age group and was statistically significant (P<0.01) (Table 3).

**Table 3:** Distribution of age group and tooth wear.

Age	Risk Level 0 n (%)	1 n (%)	2 n (%)	3 n (%)	Total
Young adults	106 (29.1)	70 (19.2)	11 (3)	1 (0.3)	188 (51.6)
Middle-Aged adults	27 (7.4)	55 (15.1)	37 (10.2)	3 (0.8)	122 (33.5)
Old adults	5 (1.4)	18 (4.9)	28 (7.7)	3 (0.8)	54 (14.8)
Total	138 (37.9)	143 (39.3)	76 (20.9)	7 (1.9)	364 (100)

The Chi-square test association between tooth wear and brushing habit showed significant association between duration of brushing (P = 0.05) and method of brushing (P<0.01) (Table 4).

**Table 4:** Association between tooth wear and brushing habits.

Brushing habits	Risk Level 0 n (%)	1 n (%)	2 n (%)	3 n (%)	Total	P
Duration of brushing						
2 minutes	82 (22.7)	71 (19.7)	40 (11.1)	3 (0.8)	196 (54.3)	
3–5 minutes	52 (14.4)	66 (18.3)	28 (7.8)	2 (0.6)	148 (41)	0.05
>5 minutes	3 (0.8)	5 (1.4)	7 (1.9)	2 (0.6)	17 (4.7)	
Total	137 (38)	142 (39.3)	75 (20.8)	7 (1.95)	361 (100)	
Method of brushing						
Horizontal	33 (9.1)	44 (12.2)	38 (10.5)	3 (0.8)	118 (32.7)	0.003
Circular	73 (20.2)	67 (18.6)	21 (5.8)	4 (1.1)	165 (45.7)	
Vertical	11 (3)	11 (3)	2 (0.6)	0	24 (6.6)	
Combination	20 (5.5)	20 (5.5)	14 (3.9)	0	54 (15)	
Total	137 (38)	142 (39.3)	75 (20.8)	7 (1.95)	361 (100)	

**Figure 1.**
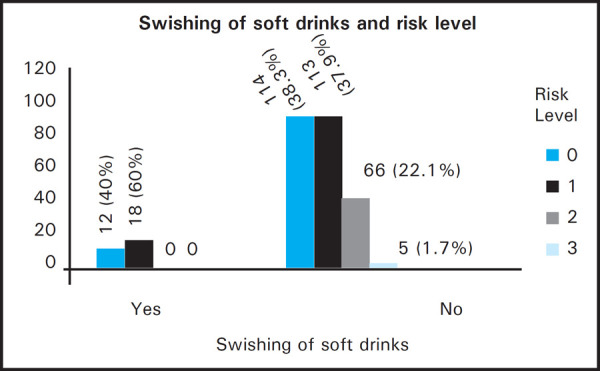
Association between tooth wear and swishing of soft drinks.

There was no association between tooth wear and dietary habits like vegetarian or non-vegetarian diet, frequency of taking sour food, frequency of taking soft drinks and frequency of taking hard food. However, there was increased tooth wear in individuals who swished soft drink in mouth before swallowing (P<0.01) (Figure 1).

**Figure 2.**
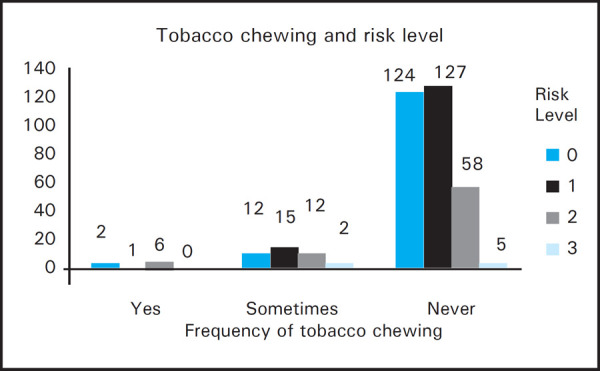
Association between tooth wear and tobacco chewing.

No effect of vomiting and reflux was seen on tooth wear. Adverse habit like drinking and parafunctional habit had no effect on tooth wear but significant association was observed between tooth wear and tobacco chewing (P=0.02) (Figure2).

## DISCUSSION

Tooth wear has increasingly become recognized as an important dental condition.^4^ This cross-sectional study was used to determine the prevalence of tooth wear in different age groups and its associated risk factors. A pretested valid questionnaire was used with questions on demographics, brushing habits, dietary habits, general health and life style. This study used BEWE, a valid and reliable index, which provides clinicians with risk indicators of a patient's level of tooth wear and may help to guide clinical management.^5^ The prevalence of tooth wear was 60.1% which was similar to studies done in France, Europe and China.^6–8^ Other studies showed a lower prevalence of 3% to 43%.^9,10^ Change in dietary pattern and increase in consumption of soft drinks may be one of the reasons of higher tooth wear prevalence. Tooth wear increased significant with increasing age group which was in agreement with other observations.^8–12^ The reason may be an underlying natural process, occurring during life, which results in a gradual increase in tooth wear.^4^ There was no gender difference in prevalence of tooth wear which was similar to other study findings.^8,9,12,13^

This study showed no relation of tooth wear and frequency of tooth brushing which was in agreement with study of Sadaf et al, Sahel et al. and Bartlett et al.^7,12,14^ However, Wet et al. and Sunny et al. showed a significant association.^8,12^ An in vitro study demonstrated no association between duration of brushing which is comparable to this study.^15^ Similar to a Nigerian study, no relationship was seen between brushing aid (tooth brush, datiwan and finger) and tooth wear.^16^ This study demonstrated that horizontal tooth brushing method caused more tooth wear which was similar to other studies.^11,16^ Therefore, it can be advised not to use horizontal or scrubbing motion and encourage circular brushing which had least tooth wear.

There was no difference in prevalence of tooth wear in vegetarian and non-vegetarian individuals. Likewise, a study done on vegetarian and non-vegetarian children showed no significant difference.^17^ The frequency of intake of hard food increased the risk of tooth wear, however, it was not significant. This study showed no increase in tooth wear in patients taking acidic food and soft drinks (coke, fanta, juice, etc.) frequently which was similar to study done in Chinese adults and Nigerians.^8,12^ In contrast, studies done in China, Poland and Europe showed a significant association.^7,8,18^ Swishing or pooling of soft drinks before swallowing increased the risk of tooth wear as shown by Wei et al. and Sunny et al.^8,16^ This may be because, in the initial stage, enamel may be softened by acid and when the attack is persistent, it causes low tooth surface pH and is eventually etched away.^19^

Intrinsic acid in cases of reflux, anorexia, bulimia or rumination can also cause erosive tooth wear and has been demonstrated by other studies.^7,8,11,18^ Though this study also showed no significant association of tooth wear and reflux, patients with these problems must be referred for medical management and prevention of erosion. No association was observed in patients with or without parafunctional habits (clenching, grinding, bruxism), which was in agreement with previous studies.^8,13,14^ In alcoholics, no significant tooth wear was observed, nevertheless, it was common in patients who had a habit of chewing tobacco which was same as two studies done in India.^9,20^ The rationale for this may be, when tobacco products containing abrasive silica are mixed with saliva and chewed, an abrasive paste is formed that over times can wear down the teeth which increases with the frequency and duration of chewing tobacco.^20^

Biological, behavioral and chemical factors are interacting with the tooth surface, which over time, may either wear it away, or indeed protect it.^19^ All these factors have not been taken into consideration in this study and future longitudinal studies must be conducted to observe their effect. Moreover, the interplay of all these factors is crucial and helps explain why some individuals exhibit more erosion than others, even if they are exposed to exactly the same acid challenge in their diets.^19^ Therefore, it is necessary to take detailed history on risk indicators for prevention, diagnosis and management. The dentists must be all aware of these factors and must be actively involved in patient education, prevention and management. Furthermore, government policies must be made regarding increase in tax on tobacco and acidic beverages so as to discourage the consumption of these products. At national level, regular oral health awareness programs can be conducted to improve the oral hygiene habits of Nepalese, which in the long run will decrease prevalence of tooth wear along with other oral diseases.

## CONCLUSIONS

Prevalence of tooth wear in the study population is high. A significant association was observed between tooth wear and age group, duration of brushing, method of brushing, swishing of soft drinks before swallowing, reflux and tobacco chewing.
